# Supplementary material for: Pharmacological Inhibition of Myostatin in a Mouse Model of Typical Nemaline Myopathy Increases Muscle Size and Force
Source: Int J Mol Sci. 2023 Oct 12;24(20):15124. doi: 10.3390/ijms242015124 (PMC10606666; doi:10.3390/ijms242015124)
Supplement: Supplementary file 1 [file ijms-24-15124-s001.zip › Supplemental Figure 2023-09-26JL.pptx]

## Slide 1
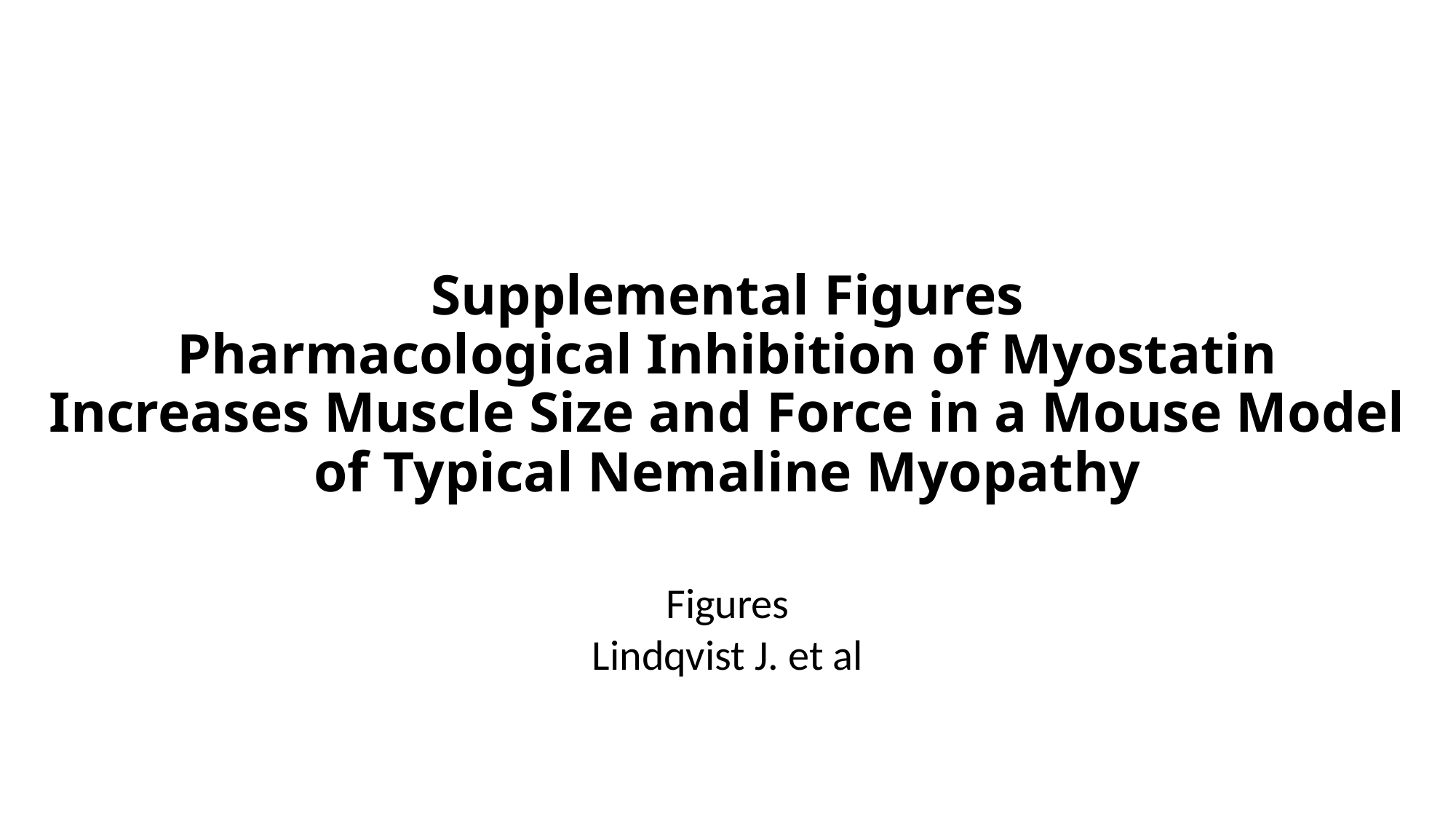

# Supplemental Figures Pharmacological Inhibition of Myostatin Increases Muscle Size and Force in a Mouse Model of Typical Nemaline Myopathy
Figures
Lindqvist J. et al

## Slide 2
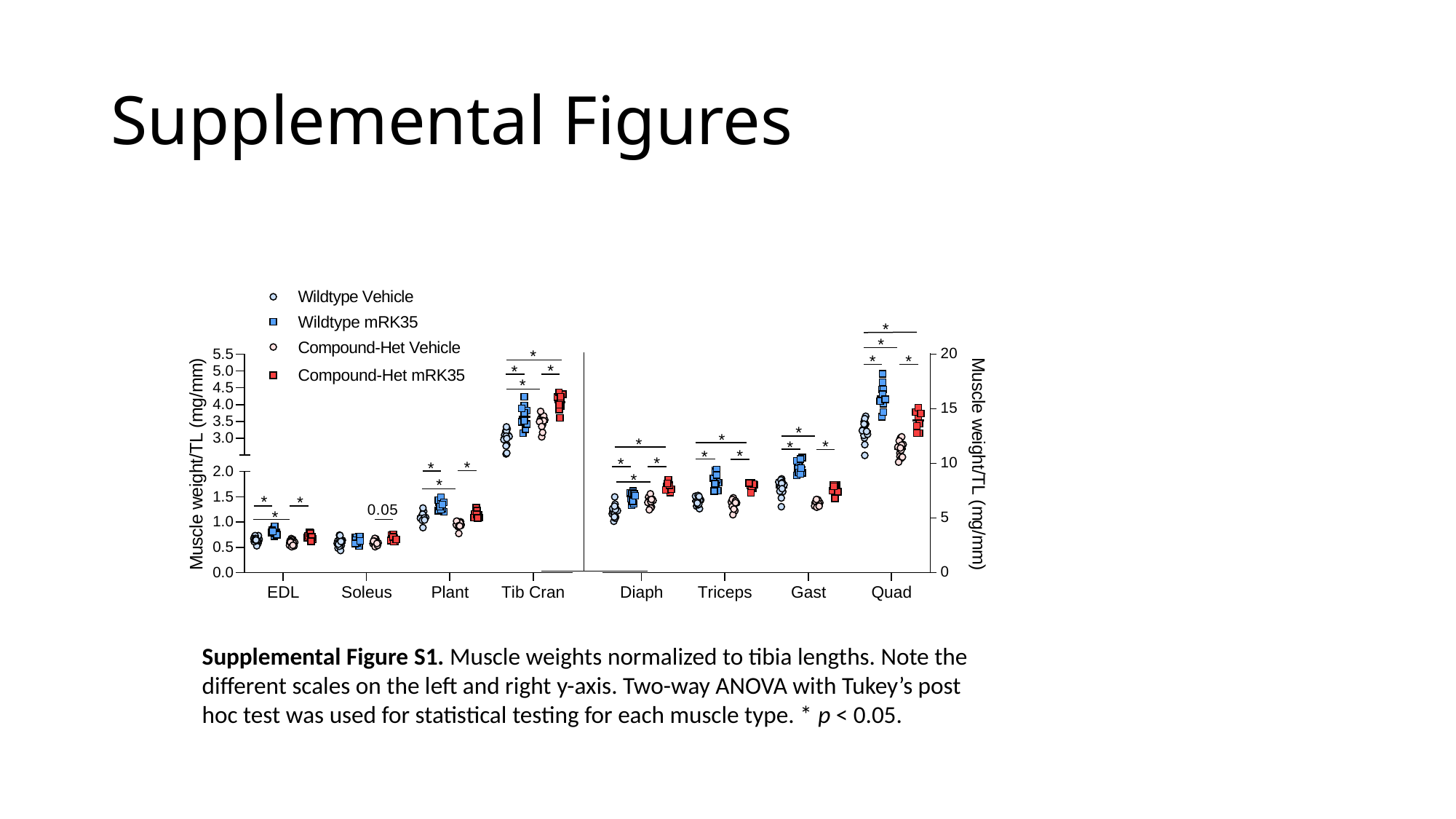

# Supplemental Figures
Supplemental Figure S1. Muscle weights normalized to tibia lengths. Note the different scales on the left and right y-axis. Two-way ANOVA with Tukey’s post hoc test was used for statistical testing for each muscle type. * p < 0.05.

## Slide 3
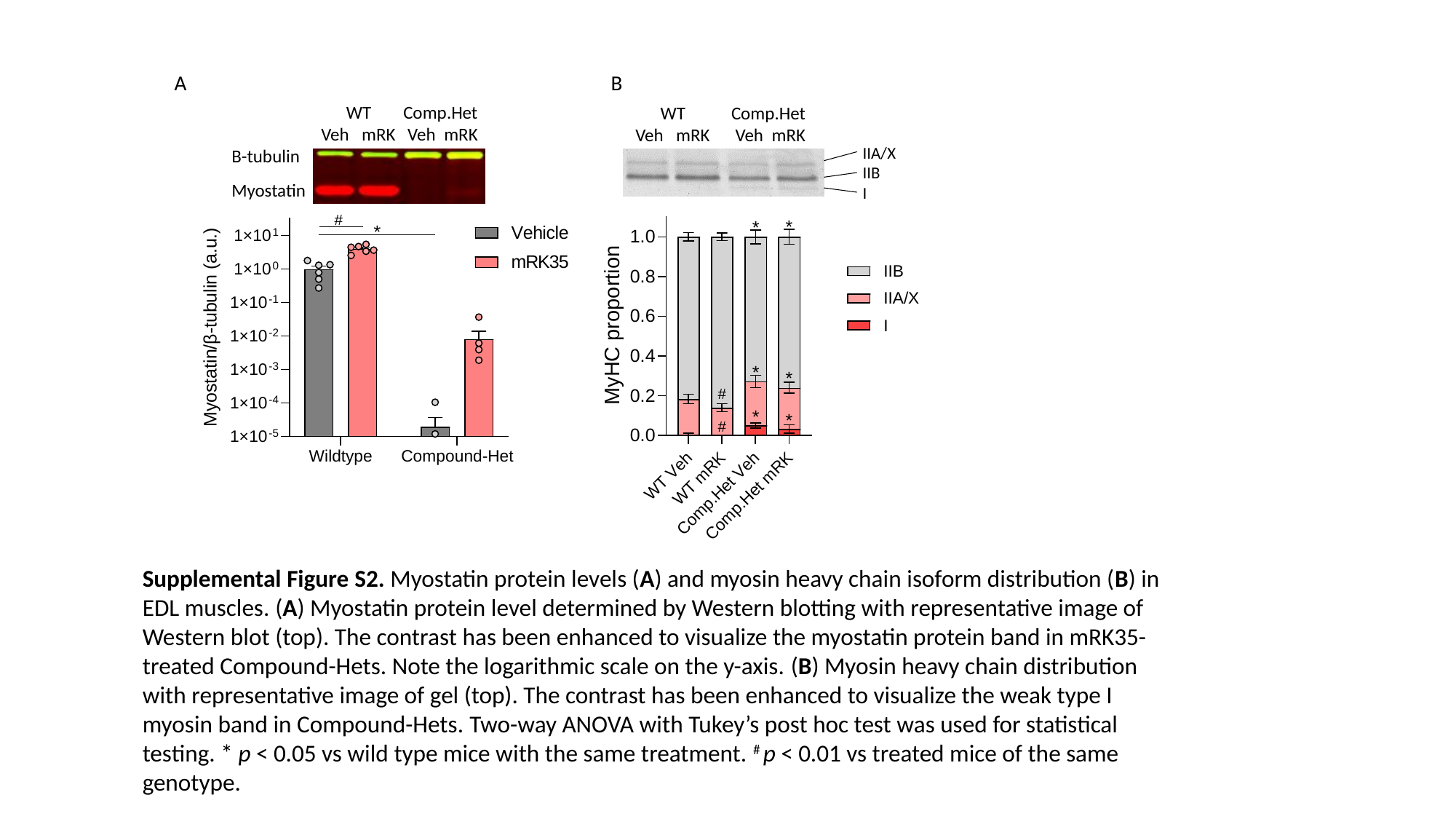

A				B
Comp.Het
 Veh mRK
 WT
Veh mRK
 WT
Veh mRK
Comp.Het
 Veh mRK
Β-tubulin
Myostatin
IIA/X
IIB
I
Supplemental Figure S2. Myostatin protein levels (A) and myosin heavy chain isoform distribution (B) in EDL muscles. (A) Myostatin protein level determined by Western blotting with representative image of Western blot (top). The contrast has been enhanced to visualize the myostatin protein band in mRK35-treated Compound-Hets. Note the logarithmic scale on the y-axis. (B) Myosin heavy chain distribution with representative image of gel (top). The contrast has been enhanced to visualize the weak type I myosin band in Compound-Hets. Two-way ANOVA with Tukey’s post hoc test was used for statistical testing. * p < 0.05 vs wild type mice with the same treatment. # p < 0.01 vs treated mice of the same genotype.

## Slide 4
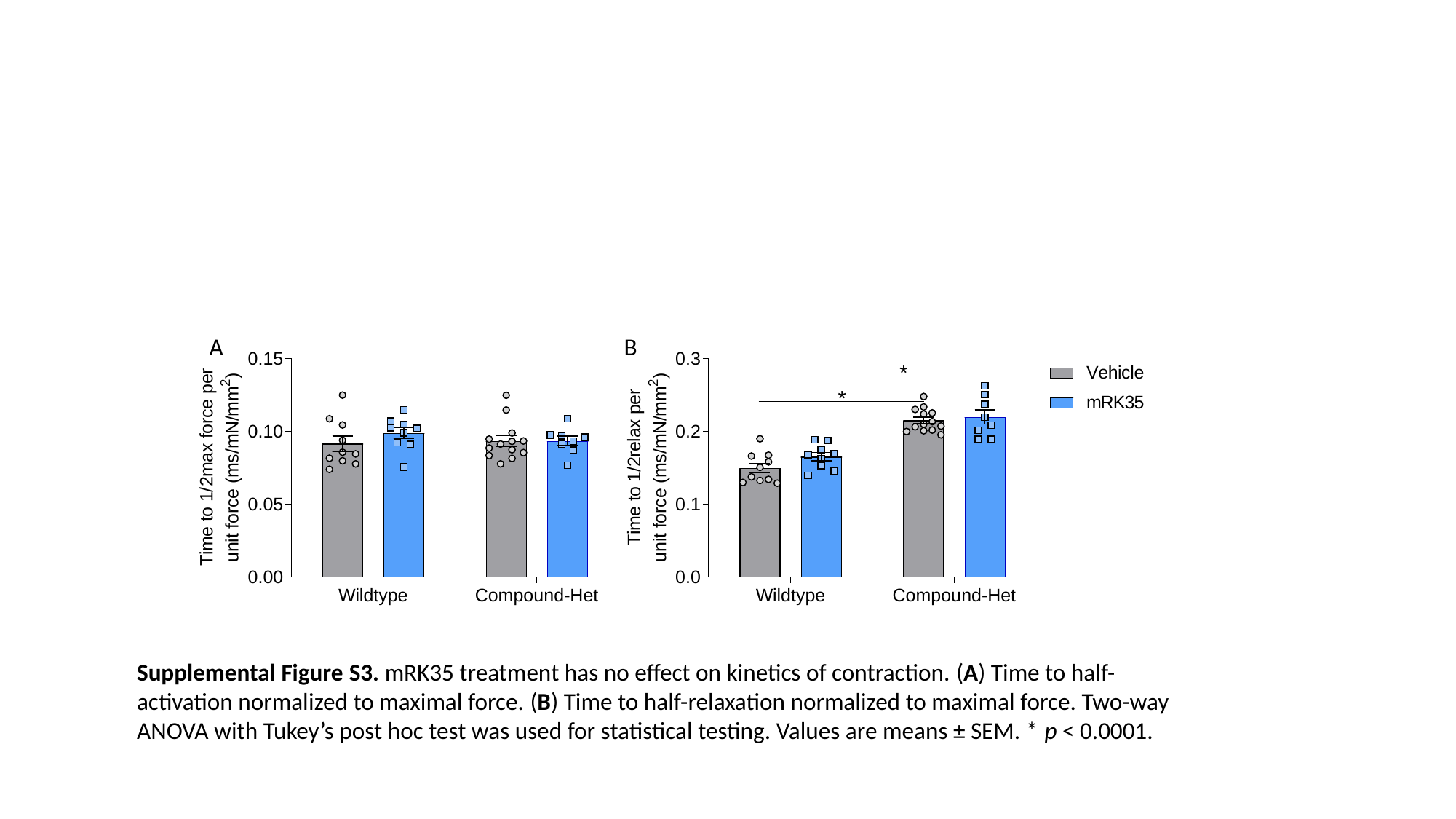

#
A			 B
Supplemental Figure S3. mRK35 treatment has no effect on kinetics of contraction. (A) Time to half-activation normalized to maximal force. (B) Time to half-relaxation normalized to maximal force. Two-way ANOVA with Tukey’s post hoc test was used for statistical testing. Values are means ± SEM. * p < 0.0001.

## Slide 5
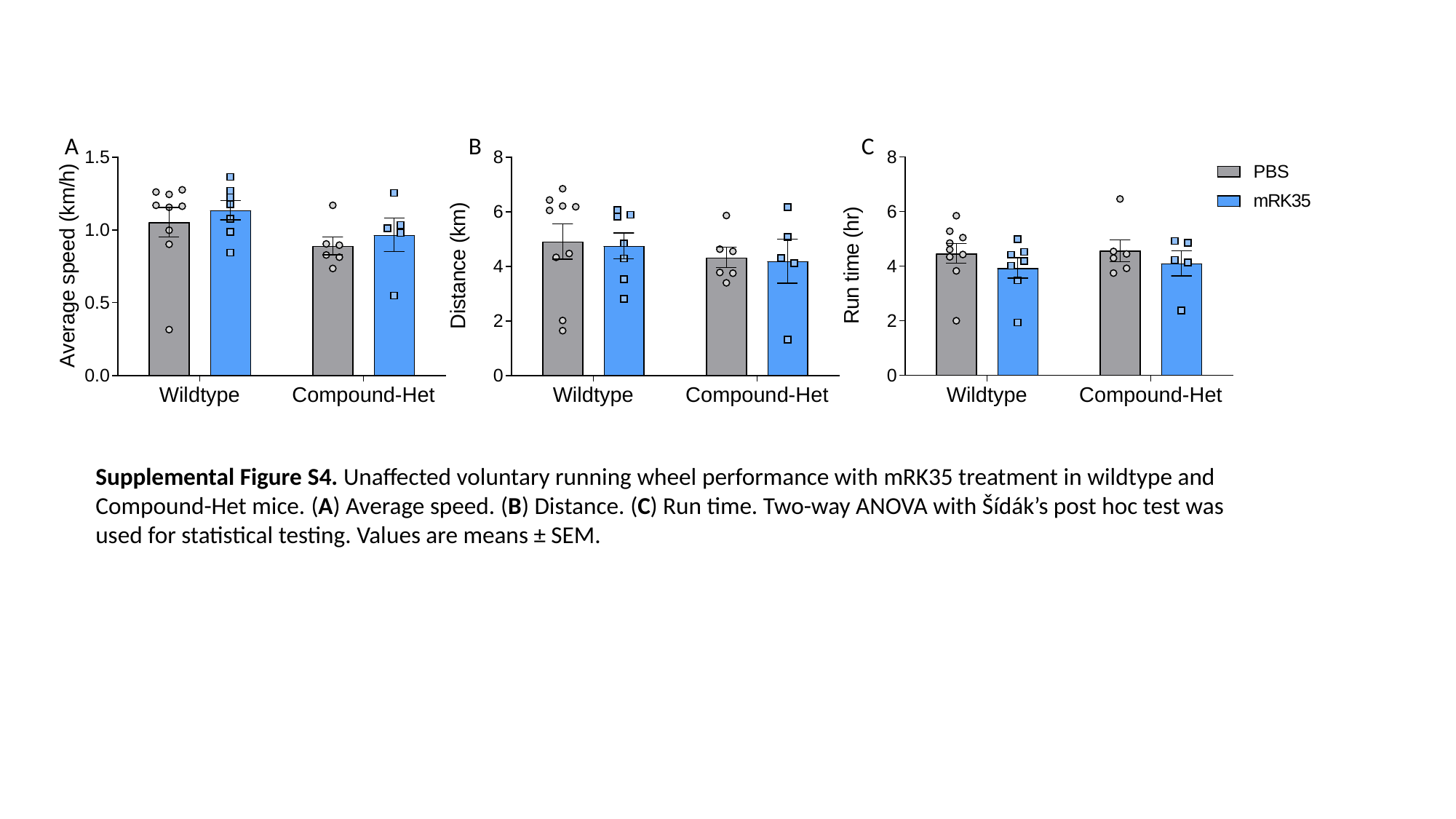

A			 B				 C
Supplemental Figure S4. Unaffected voluntary running wheel performance with mRK35 treatment in wildtype and Compound-Het mice. (A) Average speed. (B) Distance. (C) Run time. Two-way ANOVA with Šídák’s post hoc test was used for statistical testing. Values are means ± SEM.
